# Metabolite biomarker discovery for human gastric cancer using dried blood spot mass spectrometry metabolomic approach

**DOI:** 10.1038/s41598-022-19061-3

**Published:** 2022-08-27

**Authors:** Xue Wu, Huaixuan Ao, Hui Gao, Zhitu Zhu

**Affiliations:** 1 Guizhou University of Traditional Chinese Medicine, Guiyang, 550025 Guizhou China; 2 The Second Affiliated Hospital of Guizhou University of Traditional Chinese Medicine, Guiyang, 550003 Guizhou China; 3 Research Centre for Southern Deer at Guizhou University of Traditional Chinese Medicine, Guiyang, China; 4 Research Centre for Medical Data at Guizhou University of Traditional Chinese Medicine, Guiyang, China; 5 The First Affiliated Hospital of Jinzhou Medical University, Jinzhou, 121000 Liaoning China

**Keywords:** Cancer, Biomarkers, Diseases, Oncology

## Abstract

As one of the most common malignancies, gastric cancer (GC) is the third leading cause of cancer-related deaths in China. GC is asymptomatic in early stages, and the majority of GC mortality is due to delayed symptoms. It is an urgent task to find reliable biomarkers for the identification of GC in order to improve outcomes. A combination of dried blood spot sampling and direct infusion mass spectrometry (MS) technology was used to measure blood metabolic profiles for 166 patients with GC and 183 healthy individuals, and 93 metabolites including amino acids, carnitine/acylcarnitines and their derivatives, and related ratios were quantified. Multiple algorithms were used to characterize the changes of metabolic profiles in patients with GC compared to healthy individuals. A biomarker panel was identified in training set, and assessed by tenfold cross-validation and external test data set. After systematic selection of 93 metabolites, a biomarker panel consisting of Ala, Arg, Gly, Orn, Tyr/Cit, Val/Phe, C4-OH, C5/C3, C10:2 shows the potential to distinguish patients with GC from healthy individuals in tenfold cross-validation model (sensitivity: 0.8750, specificity: 0.9006) and test set (sensitivity: 0.9545, specificity: 0.8636). This metabolomic analysis makes contribution to the identification of disease-associated biomarkers and to the development of new diagnostic tools for patients with GC.

## Introduction

As one of the most common malignancies, gastric cancer (GC) is the third cause of cancer-related deaths in China^1^. According to GLOBOCAN 2018 data, around 1,034,000 new cases and more than 782,000 deaths occurred for GC in 2018^2^. GC is a multifactorial and multistep process, beginning with active chronic gastritis caused by *Helicobacter pylori* infection^3^. It is often described as a stepwise progression from non-active gastritis via chronic active gastritis into precursor lesions of GC and finally GC^4,5^. Most GC is adenocarcinoma, which derives from glandular epithelium of gastric mucosa^4^. However, GC is asymptomatic in early stages, and the majority of GC mortality is due to the delayed symptoms. It has been found that 5-year overall survival rate for patients with GC diagnosed at advanced stages is reduced down to 20%^6^. Among screening methods for early detection of GC, endoscopy as a sensitive method is most commonly used^7^, nevertheless, the risk of complications and patient discomfort limit its wide use. Furthermore, despite traditional circulating biomarkers of cancer were achieved, the diagnostic efficacy was not satisfactory for patients with GC due to their low sensitivity^8^. Because of that, finding reliable biomarkers for disease identification is of highest interest to improve outcomes for patients with GC.

Investigating the quantity or type of molecules in organisms via metabolomics can provide better understanding as to the biochemical status in a system or indicate the changes that have occurred within the metabolome^9,10^. Metabolomics technology can aid in cancer discovery and in building cancer diagnostic tools, and can provide opportunity to understand the molecular mechanism. Metabolic changes in blood are the key events in the development of carcinoma, which could be characterized by mapping global metabolic profiles, and this analytic technique has been used to interpret possible mechanisms and to identify novel metabolic biomarkers for GC^11,12^. Liquid chromatography mass spectrometry (LC–MS), one of the most commonly used platforms in metabolomic studies, can be applied to detect biomolecules for its peak resolution, high sensitivity, and sufficient reproducibility^13^. It has been found that the levels of 16 metabolites detected by LC–MS were altered in patients with GC compared to healthy control group, involving in Gly, Ala, Pro and hexadecanoic acid, which showed potential for developing biomarkers and therapeutic interventions for GC^14^. Another study showed that the ratio of kynurenine/tryptophan was associated with observed metabolic changes in patients with GC, and the monitoring of tryptophan metabolites could be used to identify potential biomarkers for GC^11^. However, both of them were relatively small-sized studies, and further clinical sample analysis is still needed for patients with GC. Besides, few reports were available in characterizing metabolic profiles of amino acids and carnitine/acylcarnitines for patients with GC. Dried blood spot (DBS) sampling is a microvolume sampling technique involving the collection of blood samples by heel or finger puncture. It as compared to conventional whole blood sampling has relatively high stability, requires a smaller blood volume, offers a simpler storage and easier transfer, reduces infection risk by infectious pathogens, and can be as an alternative method to metabolomics study^15,16^. The combination of DBS and MS can provide a high-throughput, reliable and stable determination for a broad array of analytes, which can be satisfactorily used to select high specificity and sensitivity biomarkers to some kinds of diseases^17,18^. In the present study, a combination of DBS sampling and MS was utilized to detect biomarkers based on altered levels of amino acids and carnitine/acylcarnitines in patients with GC compared to normal individuals. Nine parameters including 4 amino acids, 2 acylcarnitines and 3 related ratios were detected as potential biomarkers for GC in training set, which were further used to build prediction model for distinguishing patients with GC from healthy individuals. Hence, the changes in some amino acid and carnitine/acylcarnitine levels might indicate the existence of GC invasion, and the study’s findings suggest new insights into GC detection.

## Materials and methods

### Study design and participants

In this study, a total of 349 participants, including 183 healthy controls (HC) and 166 patients with GC, were enrolled between 2015 and 2019 from the First Affiliated Hospital of Jinzhou Medical University. Among all participants, 161 healthy individuals and 144 patients with GC were defined as training set. Other participants (22 healthy individuals and 22 patients with GC) were used as test set. Potential biomarker selection and prediction model building were performed in the training set. The external test set was used to evaluate biomarker candidates. The clinicopathologic characteristics of the whole participants were shown in Table 1. As shown in Table 1, the stages for all patients with GC were determined according to TNM (tumor-node-metastasis): stage I, 87 patients; stage II, 45 patients; stage III, 25 patients; stage IV, 9 patients. There were no significant differences identified in gender, age, BMI, blood pressure between HC and GC groups in training set and test set. The individuals with diabetes, hypertension, cardiovascular disease, tumor, infection or other diseases that can influence biological indicators or gastric function were excluded in HC group. The exclusion criteria for GC group were: (1) metabolic diseases or other digestive diseases; (2) severe liver, kidney, heart, lung, and nervous and mental diseases; (3) other types of malignant diseases and acute diseases. All the subjects with missing data were excluded. The project was approved by Ethics Committee of the First Affiliated Hospital of Jinzhou Medical University. Written informed consent was obtained from each participants. This study was conducted in accordance with tenets of the Declaration of Helsinki, and followed relevant Ethics Committee of the First Affiliated Hospital of Jinzhou Medical University guidelines and regulations.

**Table 1:** Clinicopathologic characteristics of the whole participants.

Characteristics	Training set			Test set		
	HC	GC	*p*-value	HC	GC	*p*-value
Total number	161	144		22	22	
Male	104	99	0.4427	12	12	1.0000
Female	57	45		10	10	
Age (mean, sd)	55.9627 ± 11.1147	58.1389 ± 9.7798	0.1360	55.9091 ± 8.6845	56.0909 ± 8.0647	0.9430
Weight (mean, sd)	64.5404 ± 8.4609	63.7986 ± 7.9029	0.3216	64.9545 ± 7.5181	62.6818 ± 9.9350	0.3971
Height (mean, sd)	168.5963 ± 8.6822	169.3264 ± 8.3656	0.4193	167.9091 ± 8.1761	167.2273 ± 8.4173	0.7865
BMI (mean, sd)	22.6560 ± 2.0273	22.2415 ± 2.1597	0.0920	23.0368 ± 2.0542	22.4045 ± 3.0318	0.5416
Systolic	124.7019 ± 11.0152	125.7778 ± 8.0056	0.6186	122.8636 ± 8.5149	124.5909 ± 10.9225	0.5617
**Diastolic**	75.6770 ± 9.4846	74.4375 ± 8.1599	0.1732	72.8636 ± 9.6525	72.1364 ± 12.1275	0.4704
I		75			12	
II		39			6	
III		22			3	
IV		8			1	

HC, healthy control; GC, gastric cancer; BMI: body-mass index.

### Blood sample collection and pretreatment

Labeled amino acid and relevant carnitine/acylcarnitine internal standards were mixed with pure methanol, individually. Stock solutions were prepared by the mixture of these dissolved isotope standards, and stored at 4 °C. The 100-fold dilution of stock solution was used as working solution. In quality control (QC) process, a pooled QC sample was obtained by the mixture of equal volumes (10 μL) from all collected samples.

Blood samples were collected after an overnight fasting for each participants in order to eliminate the disturbance of diet. DBS samples were collected by fingertip puncture. After wiping off the first drop of blood, 3–5 drops were collected onto a DBS card. A disc of 3 mm diameter was punched from a DBS card. The collected discs were put into Millipore MultiScreen HV 96-well plate (Millipore, Billerica, MA, USA) aimed at extracting metabolites. A working solution of 100 μL was added into a well containing a DBS disc. After 20 min gentle shaking, the plate was centrifuged at 1500 rpm for 2 min and, afterwards, the filtrate was collected into a new flat-bottom 96-well plate. In order to monitor the stability of MS analysis, 2 low-level and 2 high-level QC sample solutions were randomly put into 4 blank wells. The filtrate and QC solution were dried in pure nitrogen gas flow at 50 °C, and then these samples were derivatized with 60 μL mixture of acetyl chloride/1-butanol (10:90, v/v) at 65 °C for 20 min. After derivatized solution dried again, 100 μL mobile phase solution was mixed with each dried sample for the following metabolomics analysis.

### Metabolomics analysis

The direct injection MS was used for quantitative metabolomic analysis on an AB Sciex 4000 QTrap system (AB Sciex, Framingham, MA) coupled with an electrospray ionization source, and the MS analysis was conducted under positive mode. A sample volume of 20 μL was injected into the system. The 80% acetonitrile aqueous was used as mobile phase. An initial flow rate was set to be 0.2 mL/min. Flow rate was decreased to 0.01 mL/min within 0.08 min, and remained stable until 1.5 min. Subsequently, the flow rate reverted back to 0.2 mL/min within 0.01 min, and maintained for 0.5 min. MS parameters were set as follows: ion spray voltage 4.5 kV, curtain gas pressure 20 psi, auxiliary gas temperature 350 °C. Sheath and auxiliary gas pressure was set at 35 psi. The scan modes and scan parameters were referred to previous report^19^. Analyst 1.6.0 software (AB Sciex) was applied to control system, align spectrum, and collect MS data. ChemoView 2.0.2 (AB Sciex) was used for absolute quantification purposes.

### Data analysis

A multivariate analysis for metabolomics data was performed using SIMCA-P 12.0 software (Umetrics AB, Umea, Sweden). A principal component analysis (PCA) was used to supervise holistic metabolome alterations between patients with GC and healthy individuals and to inspect the stability of this study. In addition, a partial least squares discriminant analysis (PLS-DA) was applied to differentiate patients with GC from healthy individuals and to determine the important variables contributing to this classification based on variable importance in projection (VIP) values. Subsequently, a permutation test was used to evaluate the risk of over-fitting for PLS-DA model. T-test statistical analysis was used to identify the differential metabolites between HC and GC groups for parametric variables. Wilcoxon–Mann–Whitney test was performed for nonparametric variables. Benjamini–Hochberg false discovery rate (FDR) was used to adjust *p*-values for multiple hypothesis testing. Volcano plots were generated to screen important variables (VIP > 1, fold change (FC) > 1.2 or < − 1.2, adjusted *p*-value < 0.05) in GC group compared to HC group. In order to further investigate metabolite changes in GC group compared to HC group, significance analysis of microarrays (SAM) method was performed. Ultimately, potential biomarkers were selected by a stepwise selection method. These selected potential biomarkers were included to build a binary logistic regression model for distinguishing patients with GC from healthy individuals. The performance of this model was assessed by tenfold cross validation and external test set. Receiver-operating characteristic (ROC) curve was created to measure the ability of potential biomarkers to discriminate between patients with GC and healthy individuals. Statistical analysis was performed using SAS software. Online software MetaboAnalyst 5.0 was used for pathway analysis based on differential metabolites between HC and GC groups.

## Results

### Demographics of study samples

**Figure 1:**
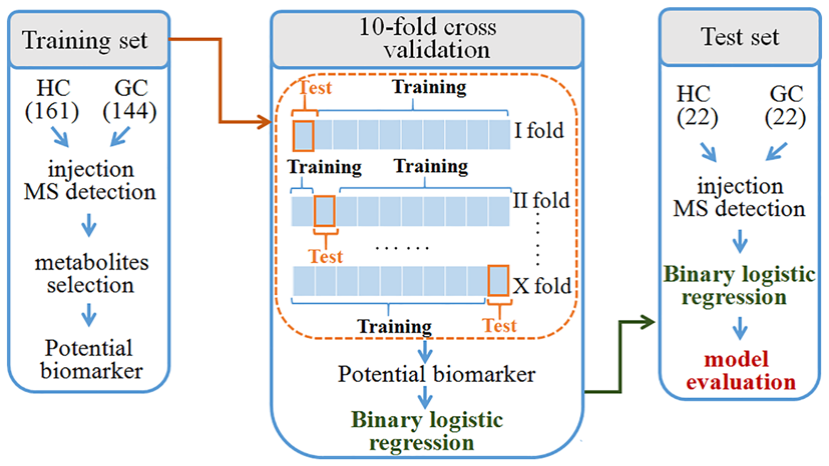
Design of the study. GC, gastric cancer; HC, healthy control.

The workflow for this study was shown in Fig. 1. A total of 305 participants, including 161 healthy individuals (mean age 55.96 ± 11.11, range 27–82 years) and 144 patients with GC (mean age 58.13 ± 9.78, range 28–85 years), were recruited as training set to define biomarker candidates. In the training set, 104 (64.60%) males and 57 (35.40%) females for healthy individuals, and 99 (68.75%) males and 45 (31.25%) females for patients with GC, were included. In order to evaluate biomarker candidates, 44 blood samples (22 healthy individuals and 22 patients with GC) were collected as test set.

### Metabolic differences between LC and HC groups

**Figure 2:**
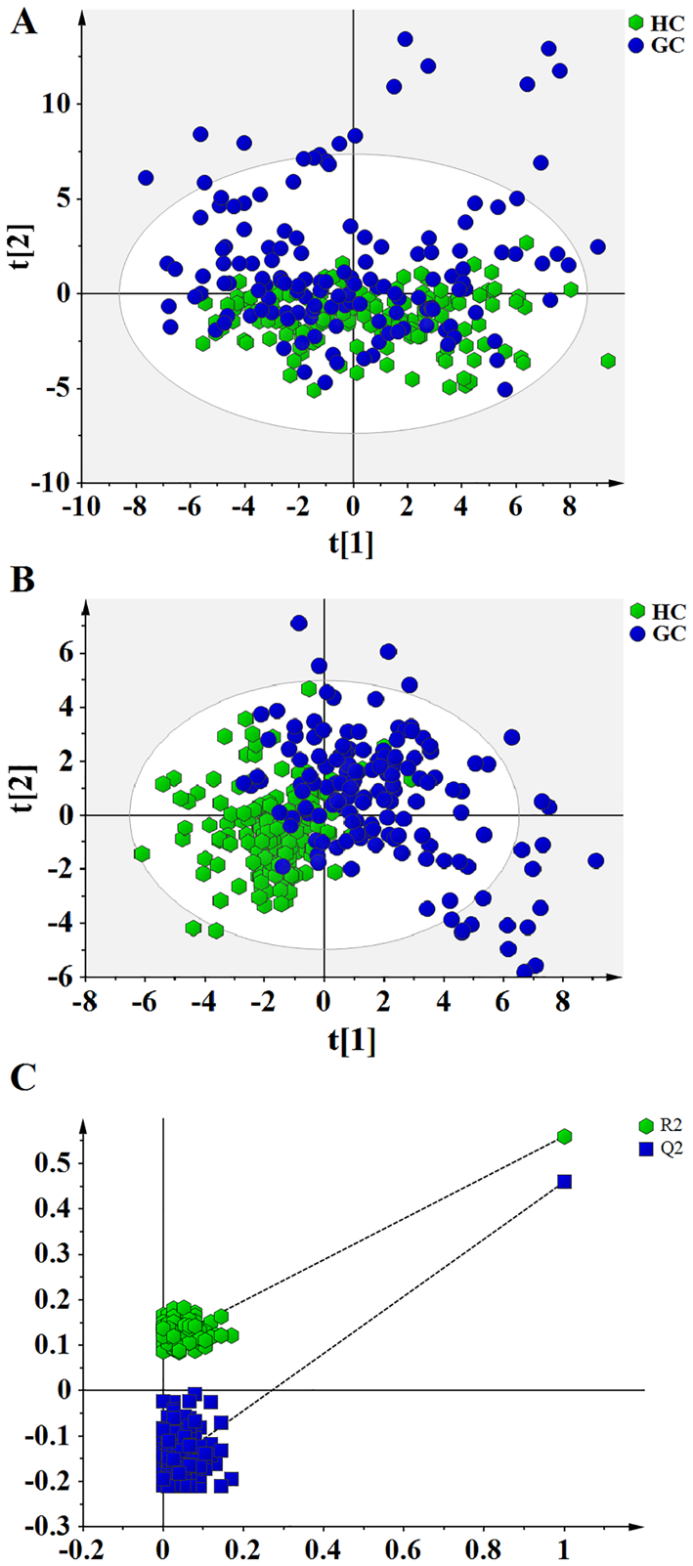
Score plots of PCA and PLS-DA analyses based on 93 metabolites for patients with GC and healthy individuals in the training set. (**A**) Score plot of PCA analysis, suggesting separating trend between GC and HC groups. The colors and shapes display the participants from different groups (healthy individuals and patients with gastric cancer). (**B**) Score plot of PLS-DA analysis, showing differential metabolic profiles in patients with GC compared to healthy individuals. (**C**) 200-times permutation test for evaluating the performance of PLS-DA model. The y-axis intercepts in test plot were R2 = (0.0, 0.109), Q2 = (0.0, -0.176).

A total of 93 variables including 23 amino acids, 26 carnitine/acylcarnitines, and 44 derived parameters and related ratios^19^ were detected from healthy participants and patients with GC for subsequent univariate and multivariate analyses. All detected variables were provided in Supplementary Table S1. Unsupervised PCA was executed for metabolomics data from blood samples of HC and GC groups in order to investigate the altered metabolites. There was a trend that GC group was separated from HC group based on 93 parameters in the training set (Fig. 2A). Furthermore, supervised PLS-DA was performed to determine the separations between HC and GC groups by all 93 variables. The PLS-DA score plot (Fig. 2B) showed the apparent separations between patients with GC and healthy individuals without over-fitting (Fig. 2C) in the training set.

### The screening of significantly differential metabolites

**Figure 3:**
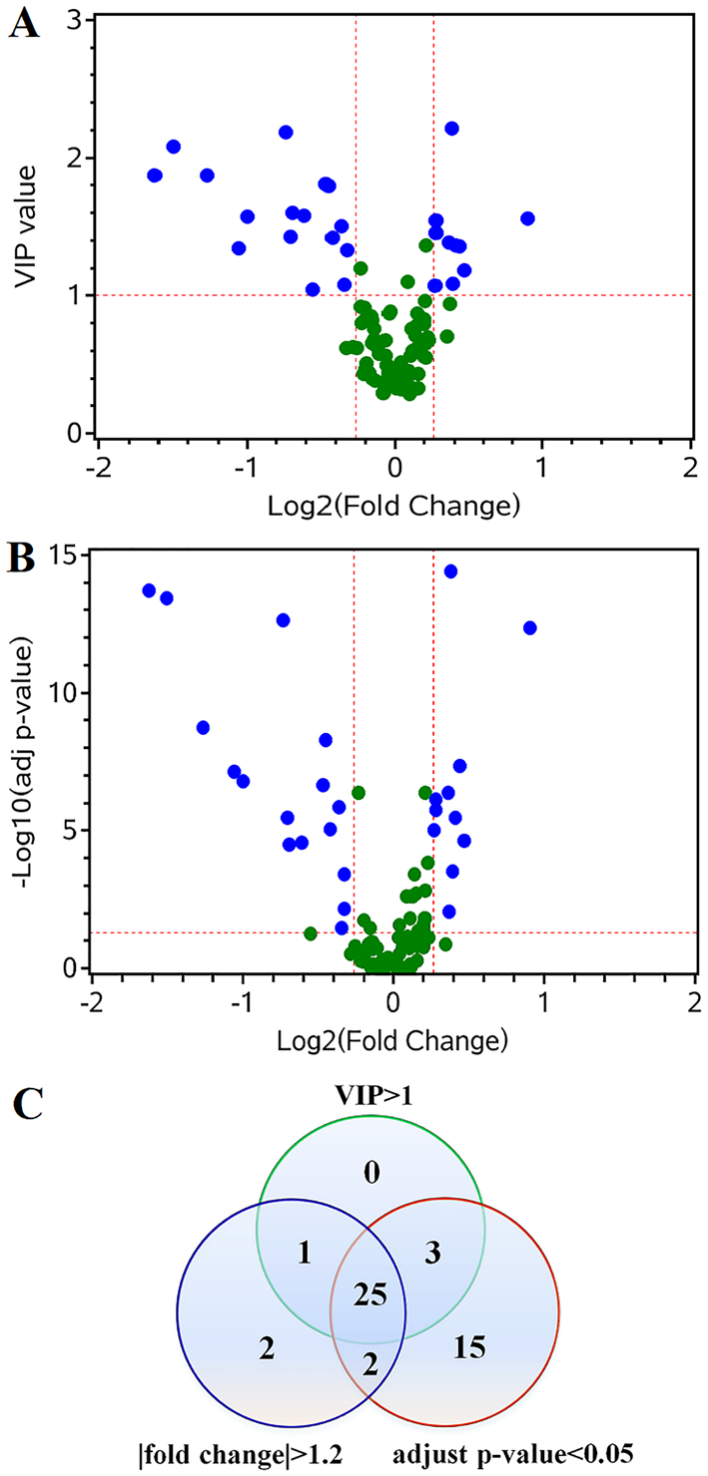
Identification of potential biomarkers for distinguishing patients with GC from healthy individuals in training set. (**A**) The plot of VIP value versus fold change (FC). The differential metabolites were defined with VIP > 1 and FC > 1.2 or < -1.2 between patients with GC and healthy individuals. The selected metabolites were colored in blue. (**B**) The plot of adjusted *p*-value versus FC. The changed metabolites were displayed with adjusted *p*-value < 0.05 and FC > 1.2 or < -1.2 in patients with GC compared to healthy individuals. (**C**) Venn diagram demonstrates differential metabolites in GC group compared with healthy group. Twenty-five differential metabolites were selected with VIP > 1 and adjusted *p*-value < 0.05 and FC > 1.2 or < − 1.2.

Systematic screening of important metabolites was executed by multiple approaches. Firstly, a total of 29 metabolites were selected with VIP > 1, which can contribute to the classification between HC and GC groups according to PLS-DA score plot (Fig. 3A). Secondly, significant differences for all metabolites were evaluated with Wilcoxon–Mann–Whitney test or t-test, and FDR was controlled in order to adjust significance levels for hypothesis testing. A total of 45 parameters were retained with adjusted *p*-value < 0.05 (Fig. 3B). Thirdly, FC was calculated, and 30 features with FC > 1.2 or < − 1.2 were significantly altered in GC group compared to HC group. Together, 25 of these metabolites with VIP > 1, adjusted *p*-value < 0.05, and FC > 1.2 or < − 1.2 exhibited significant alterations in patients with GC compared to healthy individuals in the training set (Fig. 3C). All detected variables and their adjusted *p*-value, VIP, and FC values were shown in Supplementary Table S1.

**Figure 4:**
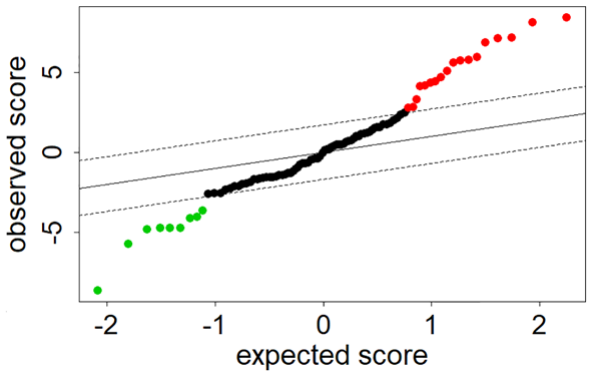
Significance analysis of microarrays for a comparison of patients with GC and healthy individuals in training set at false discovery rate of zero. The levels of 9 metabolites were significantly decreased, and 18 metabolites were significantly increased in patients with GC compared to healthy individuals.

SAM was used to further supervise and define the significant metabolite changes in patients with GC compared to healthy individuals (Fig. 4). Finally, 23 metabolites contribute to the discrepancy between the two groups (Table 2). Among these metabolites, the levels of 15 features were significantly increased and, conversely, the levels of 8 features were distinctively decreased in patients with GC compared to healthy individuals.

**Table 2:** The differential parameters between patients with GC and healthy individuals.

No	Parameters	HC (mean ± SD)	GC (mean ± SD)	Status^a^	Adjusted *p*-value
1	Asp	28.9299 ± 12.3803	44.2080 ± 31.3087	↑	< 0.0001
2	Arg	6.9661 ± 4.5362	19.7555 ± 19.3990	↑	< 0.0001
3	Gly	187.0274 ± 54.2258	255.2199 ± 112.4842	↑	< 0.0001
4	Ser	52.8482 ± 15.5849	67.8629 ± 29.7788	↑	< 0.0001
5	Orn	17.3904 ± 7.5393	53.694 ± 64.3894	↑	< 0.0001
6	C3DC	0.0681 ± 0.0462	0.1107 ± 0.0920	↑	< 0.0001
7	C4-OH	0.0620 ± 0.0338	0.1293 ± 0.1688	↑	< 0.0001
8	C18:1	0.5388 ± 0.2005	0.7210 ± 0.3814	↑	< 0.0001
9	Gly/Ala	1.1504 ± 0.5417	1.9130 ± 0.9737	↑	< 0.0001
10	Orn/Cit	0.7861 ± 0.4212	1.8922 ± 1.8845	↑	< 0.0001
11	C2/C0	0.3841 ± 0.1422	0.5307 ± 0.2511	↑	< 0.0001
12	C3DC/C10	0.5130 ± 0.4537	1.0268 ± 0.9963	↑	< 0.0001
13	C5/C3	0.0765 ± 0.0339	0.1235 ± 0.1010	↑	< 0.0001
14	C2	12.3469 ± 3.5780	15.4624 ± 8.7437	↑	0.0069
15	C5	0.1167 ± 0.0464	0.1480 ± 0.0884	↑	0.0354
16	Ala	182.8131 ± 58.0390	150.6334 ± 60.2686	↓	< 0.0001
17	Pro	492.3674 ± 166.7648	408.097 ± 231.8517	↓	< 0.0001
18	Cit/Arg	5.3826 ± 4.3734	2.8778 ± 2.9849	↓	< 0.0001
19	Met/Phe	0.4766 ± 0.1452	0.3931 ± 0.1275	↓	< 0.0001
20	Tyr/Cit	1.4338 ± 0.6711	1.0788 ± 0.5934	↓	< 0.0001
21	Val/Phe	3.7288 ± 0.9200	2.8610 ± 0.7869	↓	< 0.0001
22	C3/C2	0.1397 ± 0.0522	0.1088 ± 0.0529	↓	< 0.0001
23	C10:2	0.7606 ± 0.4541	0.5502 ± 0.3974	↓	< 0.0001

HC, healthy control; GC, gastric cancer; Asp, aspartic acid; Arg, arginine; Gly, glycine; Ser, serine; Orn, ornithine; C3DC, malonylcarnitine; C4-OH, hydroxybutyrylcarnitine; C18:1, octadecenoylcarnitine; Ala, alanine; Cit, citrulline; C2, acetylcarnitine; C0, free carnitine; C10, decanoylcarnitine; C5, isovalerylcarnitine; C3, propionylcarnitine; Pro, proline; Met, methionine; Phe, phenylalanine; Tyr, tyrosine; Val, valine; C10:2, decadienoylcarnitine.

^a^Defined as the increased (upward arrow) or decreased (downward arrow) levels of metabolites in patients with GC compared to healthy individuals.

**Figure 5:**
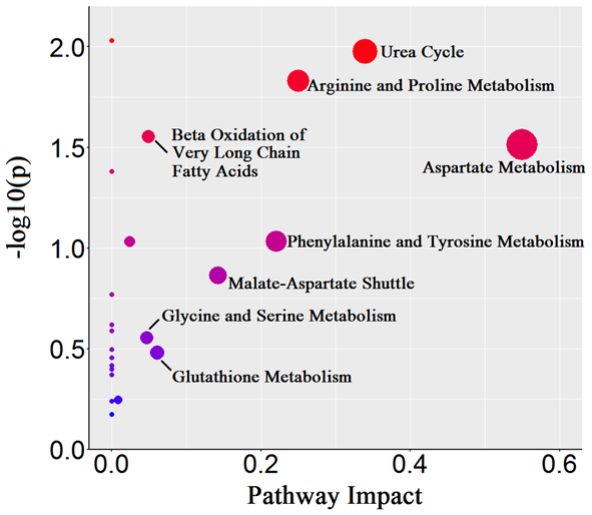
A pathway impact analysis based on differential metabolites between GC and HC groups in training set. Eight perturbed metabolic pathways were indicated for patients with GC.

To further clarify the metabolic pathways which may be affected by GC, the differential metabolites between healthy individuals and patients with GC (Table 2) were imported into MetaboAnalyst 5.0 for pathway analysis. As shown in Fig. 5, 8 metabolic pathways were highlighted focusing on amino acid metabolism, urea cycle, malate-aspartate shuttle, lipid metabolism, and so on.

### Building prediction model

**Figure 6:**
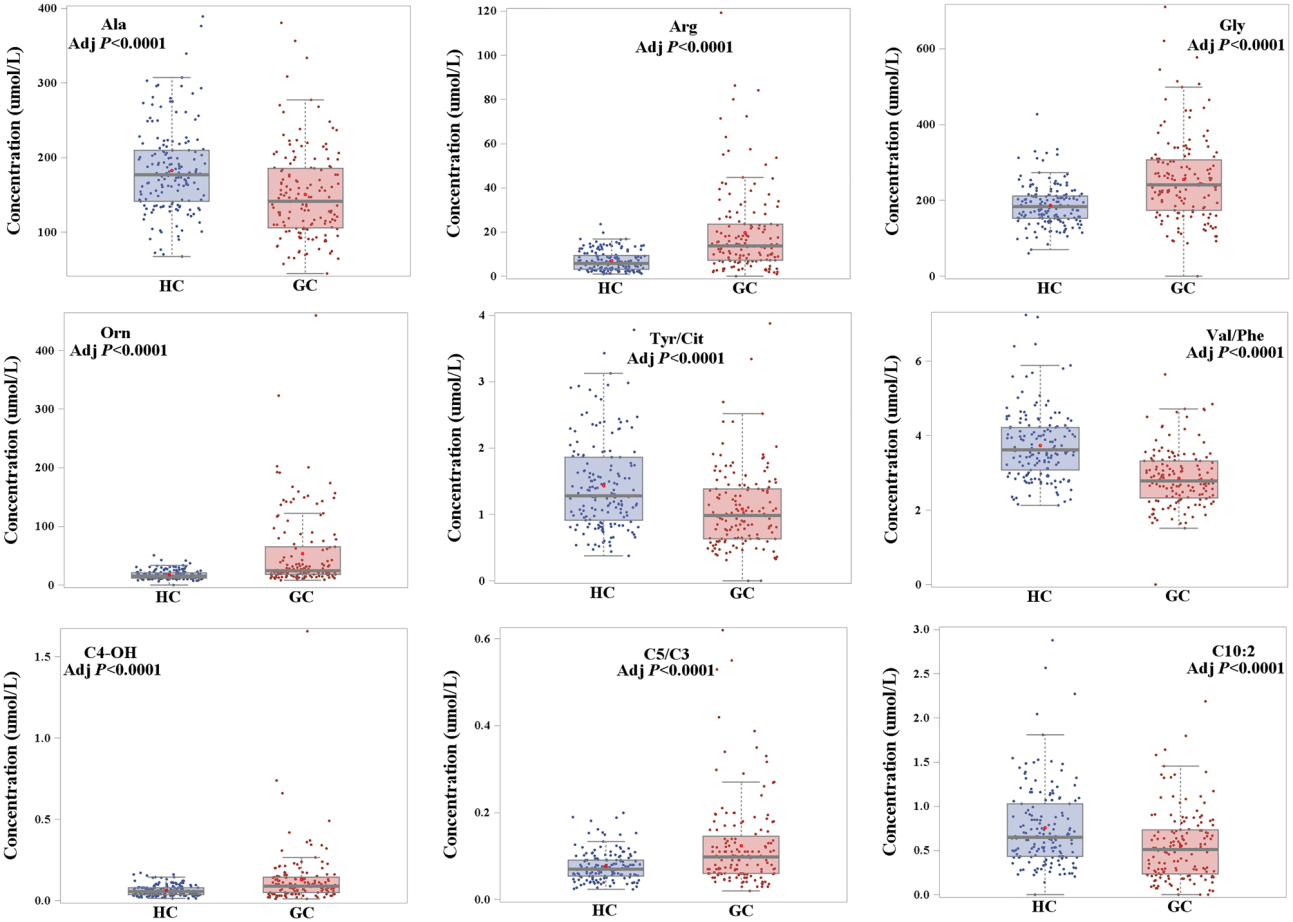
Blood concentrations for potential metabolic biomarkers contributing to the building of prediction model in training set.

**Figure 7:**
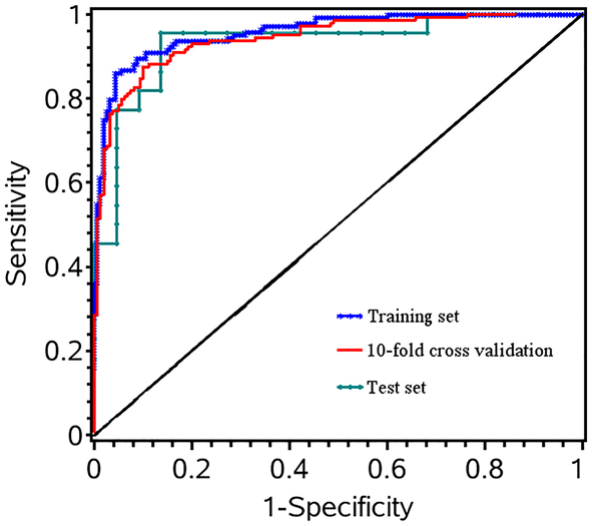
Receiver operating characteristic (ROC) curve was established to examine the performance of metabolite biomarker panel in distinguishing patients with GC from healthy individuals. ROC curve was marked with blue star for training set, red line for tenfold cross validation, and cyan dot for test set.

A stepwise logistic regression was conducted towards 23 selected metabolites (Table 2) in the training set. Finally, 9 features were identified including Ala, Arg, Gly, Orn, Tyr/Cit, Val/Phe, C4-OH, C5/C3, C10:2 (Fig. 6). A logistic regression model was developed as follows: Logit probability = 2.05 − 1.35 × Ala + 5.68 × Orn + 1.80 × Arg + 2.39 × C4-OH − 0.90 × Tyr/Cit − 0.62 × Val/Phe + 1.20 × C5/C3 − 1.35 × C10:2 + 0.76 × Gly. The diagnostic performance of this metabolic panel was evaluated by tenfold cross validation and external test set (Table 3). Furthermore, ROC curve was drawn to assess the potential of metabolic panel to distinguish between patients with GC and healthy individuals (Fig. 7). The area under ROC curve (AUC) is 0.9586 (95%CI 0.9384–0.9788) in the training set. The sensitivity and specificity were 0.8611 and 0.9565 in the training set, respectively. During the process of tenfold cross validation, all samples in the training set were randomly divided into 10 partitions in order to cross-validate the predicted model. Additionally, 44 blood samples including 22 patients with GC and 22 healthy individuals were used as test set to further assess the diagnostic potential of 9 selected metabolic biomarkers. As shown in Table 3, the AUC of 0.9438 (95%CI 0.9163–0.9714) and 0.9318 (95%CI 0.8525–1.0000) was determined in tenfold cross validation and test set, respectively. Additionally, both of sensitivity and specificity were also satisfactory in tenfold cross validation (sensitivity: 0.8750; specificity: 0.9006) and test set (sensitivity: 0.9545; specificity: 0.8636).

**Table 3:** Performance of metabolite biomarker panel for distinguishing patients with GC from healthy individuals in the training set, tenfold cross validation, and test set.

	Training set	tenfold cross validation	Test set
AUC (95%CI)	0.9586 (0.9384–0.9788)	0.9438 (0.9163–0.9714)	0.9318 (0.8525–1.0000)
Sensitivity	0.8611	0.8750	0.9545
Specificity	0.9565	0.9006	0.8636

GC, gastric cancer; AUC, area under receiver operating characteristic curve.

## Discussion

Currently, identification of novel blood biomarkers remains a pivotal goal for GC, and the limitations of modern technology for the detection and treatment of the disease emphasize the necessity of finding novel potential biomarkers. However, few biomarker candidates can be translated into clinical applications due to limited diagnostic performance or study cohorts^20^. Specific physiological or pathological conditions are able to perturb blood metabolites, which can be used as potential biological indicators in normal and pathological biological processes. Thus, the detection of perturbed small molecular metabolites can provide a powerful tool for cancer diagnosis. In the present study, a total of 349 subjects were recruited, including 183 healthy individuals and 166 patients with GC, and were divided into training set of 305 subjects and test set of 44 subjects. A combination of DBS sampling and direct injection MS analysis was performed to detect metabolite biomarkers for GC. After systematic selection, there were significant differences in the levels of 23 metabolites between patients with GC and healthy individuals (Table 2). Furthermore, independent predictors was identified by a stepwise logistic regression analysis, and a biomarker panel consisting of Ala, Arg, Gly, Orn, Tyr/Cit, Val/Phe, C4-OH, C5/C3, C10:2 was used to construct prediction model for GC.

Metabolic reprogramming was regarded as a central hallmark of cancer. The amino acid and lipid metabolic pathways were disturbed in patients with GC as revealed by differential pathway analysis (Fig. 5). Identifying how metabolism shifts in patients with cancer can contribute to disease diagnosis and prediction. Amino acids were disturbed by the imbalance in protein metabolism due to the influences of host-tumor interactions and metabolic requirements of tumor cells to specific amino acids^21^, which exhibited potential usage in improving diagnosis and detection of early-stage cancer^22^. In the present study, 23 metabolites were significantly altered with VIP > 1, adjusted *p*-value < 0.05, and FC > 1.2 or < − 1.2 in patients with GC compared to healthy individuals, involving Asp, Arg, Gly, Ser, Orn, Ala, and Pro. Interestingly, the levels of Asp, Arg, Gly, Ser, and Orn were increased in patients with GC compared to healthy individuals (Table 2). As a non-essential amino acid, Asp is the basic substrate for the synthesis of pyrimidine and purine nucleosides. Furthermore, Ser is also involved in the synthesis of purine nucleotides via Gly, and it can influence cell growth and invasion of cancer cells^23^. The increased uptake rates of Asp and Ser imply that these amino acids are needed in fueling nucleoside biosynthesis for tumor proliferation^24^. As a semi-essential amino acid, Arg is a key component in the body and involved in cell division, immune system, and hormone biosynthesis, and contributes to immunosurveillance, tumor growth and metastasis^25^, which implies that Arg is required to fuel tumor cell metabolism. Its metabolism may be influenced by the overexpressed argininosuccinate synthase 1 (ASS1) in GC^26^. The high expression of ASS1 can lead to increased NO production, which promotes gluconeogenesis via S-nitrosylation of pyruvate carboxylase and phosphoenolpyruvate carboxykinase 2. The increased gluconeogenesis may further enhance the levels of Ser and Gly in nucleotide synthesis^27^. Increased level of Arg can lead to decreased ratio of Cit/Arg, and this ratio has been found to reflect NO production^28^. Orn acts both as a substrate of ornithine decarboxylase (ODC) to produce polyamines and as a substrate of ornithine aminotransferase (OAT) to produce Pro, and all these products are involved in the promotion of cancer progression^29^. Therefore, altered levels of Orn and Pro suggested the disorders of ODC and OAT metabolism and the requirements of these amino acids in GC progression. It has been found that ratio of Orn/Cit can reflect a shift in arginine metabolism^30^. This ratio is influenced by increased level of Orn, reconfirming the altered arginine metabolism in GC.

The upregulated glycolysis in cancer metabolism, also known as the Warburg effect, promotes compensatory pathways, especially oxidation of fatty acids^31^. Carnitine/acylcarnitines, intermediates of fatty acid oxidation, are essential for fatty acid oxidation and energy metabolism, and accumulated as a consequence of metabolic defect. Considering these adaptations, carnitine pool is uniquely positioned to supervise the perturbations of carnitine/acylcarnitine metabolism, and it is useful to discover the disturbed metabolic pathways during cancer development and progression^32^. Carnitine palmitoyl transferase 1 (CPT1) is associated with metabolism of acylcarnitines by catalyzing the conversion of acyl-CoA into acylcarnitine, and controls the entry of long-chain fatty acid into mitochondrial matrix for energy production via fatty acid β-oxidation. A recent study reported that CPT1A is upregulated in patients with GC, and is involved in GC progression^33^, which may account for the distinct accumulations of acylcarnitines in GC. In addition, the expression of carnitine acetyltransferase (CrAT) has been reported to be upregulated in cancer, which may also promote alterations in carnitine metabolism^34^. In this study, the levels of 4 short-chain acylcarnitines (C2, C3DC, C4-OH, C5) and one long-chain acylcarnitine (C18:1) were increased in patients with GC compared to healthy individuals (Table 2), and the accumulations of acylcarnitine metabolites may be due to the abnormal expression of these enzymes. Ratio C2/C0 was enhanced in patients with GC, which further indicated the increased fatty acyl mitochondrial transport and β-oxidation of fatty acids in GC^35^. Furthermore, it has been reported that short-chain carnitine-acylcarnitine translocase in mitochondria and short-chain acylcarnitine levels may be related to the metabolism of branched-chain amino acids (BCAA)^36^, and changed ratio of C3/C5 implicates altered flux through BCAA metabolic pathways^37^. The abnormal levels of short-chain acylcarnitines may further influence ratio Val/Phe. Several studies reported that free carnitine and short-, medium-, and long-chain acylcarnitines were disturbed in patients with cancer, and showed potential as candidate biomarkers for the development of certain cancers^38,39^. Taken together, these findings suggested that the detection of carnitine/acylcarnitine changes may provide a promising new strategy against GC.

A high-performance biomarker panel consisting of Ala, Arg, Gly, Orn, Tyr/Cit, Val/Phe, C4-OH, C5/C3, C10:2 was identified and validated for separating patients with GC from healthy individuals, as displayed in Table 3. The tenfold cross-validation was performed to evaluate classifier performance by using the data in training set, which showed that the diagnostic performance of this biomarker panel was satisfactory (AUC: 0.9438). An independent test data set of 44 subjects, including 22 patients with GC and 22 healthy individuals, was applied to assess reliability of this metabolite biomarker panel. It showed that this biomarker panel can effectively discriminate patients with GC from healthy individuals (AUC: 0.9318). These results highlighted that the metabolite biomarker panel may act as a potential valuable tool to detect GC.

In this single-center case-control study, a combination of DBS sampling and MS was utilized for high-throughput detection of metabolites. A metabolite biomarker panel was identified with diagnostic potential for GC. Whereas, there were some limitations in this study. Firstly, since GC is considered as a stepwise progression from non-active gastritis^4,5^, we believe that this study will be more systematic when a reasonable amount of patients with gastritis can be recruited. Secondly, in this study, the detected metabolites were limited for the trade-off between coverage and cost, and more metabolites such as fatty acids will be detected in order to select more potential biomarkers. Thirdly, more patients with advanced GC will be recruited in order to perform metabolomics analysis based on the patients in different stages. Finally, a multi-institution study with a larger sample size is still required in order to further assess this study’s results.

## Conclusion

In summary, a combination of DBS sampling and direct injection MS technology was used to detect metabolites for patients with GC and healthy individuals. Results obtained displayed significantly altered metabolomic profiles in patients with GC compared to healthy individuals. A metabolite biomarker panel of Ala, Arg, Gly, Orn, Tyr/Cit, Val/Phe, C4-OH, C5/C3, C10:2 was determined as an effective tool with satisfactory sensitivity and specificity for discriminating patients with GC from healthy individuals. Therefore, we believe that these selected metabolites have potential as novel biomarkers in the detection of GC.

## Supplementary Information

Supplementary Table S1.

## Data Availability

The datasets used and/or analysed during the current study available from the corresponding author on reasonable request.

## Abbreviations

GC: Gastric cancer
HC: Healthy control
MS: Mass spectrometry
DBS: Dried blood spot
QC: Quality control
PCA: Principal component analysis
PLS-DA: Partial least squared discriminant analysis
VIP: Variable importance in projection
FDR: False discovery rate
FC: Fold change
SAM: Significance analysis of microarrays
ROC: Receiver operating characteristic
AUC: Area under ROC curve
Asp: Aspartic acid
Arg: Arginine
Gly: Glycine
Ser: Serine
Orn: Ornithine
C3DC: Malonylcarnitine
C4-OH: Hydroxybutyrylcarnitine
C18:1: Octadecenoylcarnitine
Ala: Alanine
Cit: Citrulline
C2: Acetylcarnitine
C0: Free carnitine
C10: Decanoylcarnitine
C5: Isovalerylcarnitine
C3: Propionylcarnitine
Pro: Proline
Met: Methionine
Phe: Phenylalanine
Tyr: Tyrosine
Val: Valine
C10:2: Decadienoylcarnitine
ASS1: Argininosuccinate synthase 1
ODC: Ornithine decarboxylase
OAT: Ornithine aminotransferase
CPT1: Carnitine palmitoyl transferase 1
